# Correction: *Drosophila* and human cell studies reveal a conserved role for CEBPZ, NOC2L and NOC3L in rRNA processing and tumorigenesis

**DOI:** 10.1242/jcs.264718

**Published:** 2026-02-11

**Authors:** Guglielmo Rambaldelli, Valeria Manara, Andrea Vutera Cuda, Giovanni Bertalot, Marianna Penzo, Paola Bellosta

There was an error in *J. Cell Sci.* (2025) **138**, jcs264096 (doi:10.1242/jcs.264096).

The labels for Fig. 1M were accidently swapped. Fig. 1 has been updated to show the correct labels. The corrected and original panels are shown below.

**Fig. 1M:**
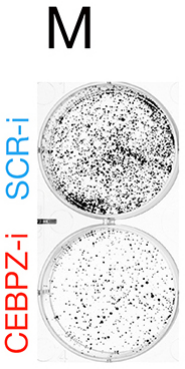
**(corrected panel). Reduction of NOC proteins disrupts rRNA processing, increases p53 expression, and reduces protein synthesis and cell growth.** (M) Representative photos of colonies at 10 days of treatment.

**Fig. 1M:**
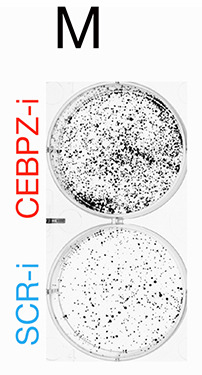
**(original panel). Reduction of NOC proteins disrupts rRNA processing, increases p53 expression, and reduces protein synthesis and cell growth.** (M) Representative photos of colonies at 10 days of treatment.

This figure has been corrected in the online and pdf versions of the paper. The authors apologise for any inconvenience caused.

