# Supplementary material for: Drosophila and human cell studies reveal a conserved role for CEBPZ, NOC2L and NOC3L in rRNA processing and tumorigenesis
Source: J Cell Sci. 2025 Sep 12;138(17):jcs264096. doi: 10.1242/jcs.264096 (PMC12450466; doi:10.1242/jcs.264096)
Supplement: Supplementary information [file joces-138-264096-s1.pdf]

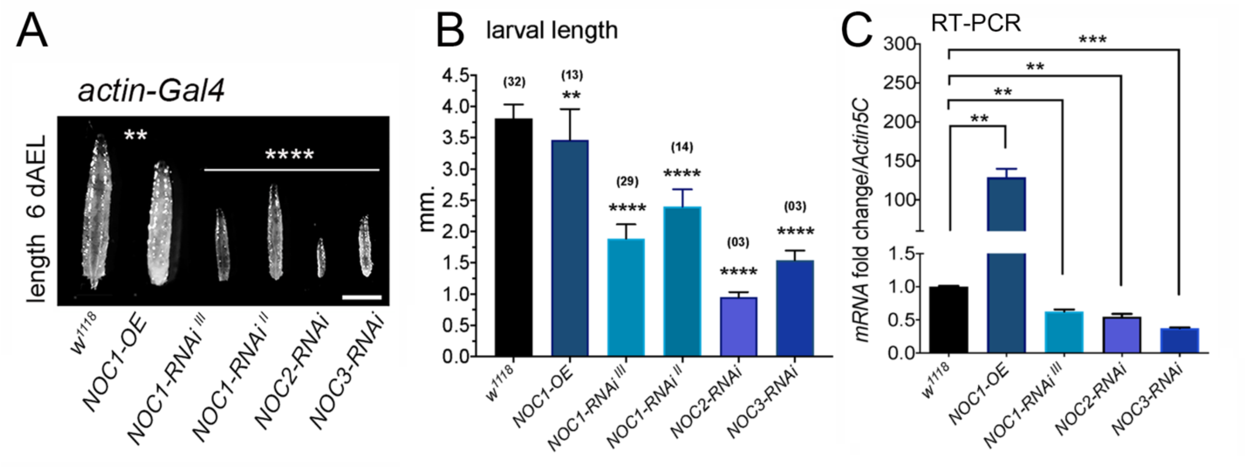

Fig. S1. Reducing NOCs affects animal growth.

(A) Photos of larvae of the indicated genotype and (B) larval length, measured at 120 hours after egg laying (AEL). The asterisks represent the *p*-values from one-way analysis of variance (ANOVA) with Tukey multiple comparisons ** = $p < 0.01$ and **** = $p < 0.0001$, and the error bars indicate the standard deviations for each genotype. In parenthesis, the number of animals analyzed is indicated. (C) qRT-PCR showing the relative amount of *NOCs mRNA* upon RNAi. *NOCs-RNAi* were ubiquitously expressed using the *actin-Gal4* promoter. RNA was extracted from whole larvae. *p*-values were calculated from the Student's *t*-test from at least two independent experiments: ** = $p < 0.01$, *** = $p < 0.001$, and **** = $p < 0.0001$; the error bars indicate the standard deviations. Note: In these experiments, the effect of NOC1-overexpression is also reported since these photos and data have been published by us in our previous paper (Destefanis et al., 2022) and are represented here to show the efficiency of the RNAi interference experiments.

Destefanis, F., Manara, V., Santarelli, S., Zola, S., Brambilla, M., Viola, G., Maragno, P., Signoria, I., Viero, G., Pasini, M.E., Penzo, M., and Bellosta, P. (2022). Reduction of nucleolar NOC1 leads to the accumulation of pre-rRNAs and induces Xrp1, affecting growth and resulting in cell competition. *J Cell Sci* 135.

Table S1. NOC proteins alignment

Human CEBPZ and Drosophila NOC1 protein sequences

```
CEBPZ human >sp|Q03701|1-1054
MAAVKEPLEFHAKRPWRPEEAVEDPDEEDEDNTSEAENGFSLEEVLRLGGTKQDYLMLAT
LDENEEVIDGGKKGAIDDLQQGELEAFIQNLNLAKYTKASLVEEDEPAEKENSSKKEVKI
PKINNKNTAESQRTSVNKVKNKNRPEPHSDENGSTTPKVKKDKQNIFEFFERQTLLLRPG
GKWYDLEYSNEYSLKPQPQDVVSKYKTLAQKLYQHEINLFKSKTNSQKGASSTWMKAIVS
SGTLGDRMAAMILLIQDDAVHTLQFVETLVNLVKKKGSKQQCLMALDTFKELLITDLLPD
NRKLRIFSQRPFDKLEQLSSGNKDSRDRRLILWYFEHQLKHLVAEFVQVLETLSHDTLVT
TKTRALTVAHELLCNKPEEEKALLVQVVNKLGDPQNRIATKASHLLETLLCKHPNMKGVV
SGEVERLLFRSNISSKAQYYAICFLNQMALSHEESELANKLITVYFCFFRTCVKKKDVES
KMLSALLTGVNRAYPYSQTGDDKVREQIDTLFKVL516HIVNFNTSVQALMLLFQVMNSQ
QTISDRYYTALYRKMLDPGLMTCSKQAMFLNLVYKSLKADIVLRRVKAFVKRLLQVTCQQ
MPPFICGALYLVSEILKAKPGLRSQLDDHPESDDEENFIDANDDEDMEKFTDADKETEIV
KKLETEETVPETDVETKKPEVASWVHFDNLKGGKQLNKYDPFSRNPLFCGAENTSLWELK
KLSVHFHPSVALFAKTILQGNYIQYSGDPLQDFTLMRFLDRFVYRNPKPHKGKENTDSVV
MQPKRKHFIKDIRHLPVNSKEFLAKEESQIPVDEVFFHRYYKKVAVKEKQKRDADEESIE
DVDDEEFEELIDTFEDDNCFSSGKDDMDFAGNVKKRTKGAKDNTLDEDSEGSDDELGNLD
DDEVSLGSMDDEEFAEVDEDGGTFMDVLDDESESVPELEVHSKVSTKKSKRKGTDDFDFA
GSFQGPRKKKRNLNDSSLFVSAEEFGHLLDENMGSKFDNIGMNAMANKDNASLKQLRWEA
ERDDWLHNRDAKSIIKKKKHFKKKRIKTTQKTKKQRK

NOC1 Dm >tr|Q9VTE6|1-1174
MPAAVATGVQFGGPPKNKKIVFDDSGEAVVKQNKKEHPQRPKFEGKEQVKKPQKIKFGED
GKAKGAKSFNKNHQKPDFANKPQRIKFGDDGEQVASKSFNQNHKNGPKPQKIKFGEDREA
VHQKPFNKNNHKHNGQKSDFANKPQKIKFTDDGEDEVTANSSNTKTEPTKKSQKIKFGDD
GESKENFKKPQRIKFDEDGAGKNVSDSDGDSDEELGDSISKKHNKYQSKIDEDEESQKKW
YHVHPDYPSTDEVLDMKENDQLELYNLCKNSFEAEKITF280NKRNPSDARWLQTALHKG
TAKDRANAGALLVTSNPLGNLEALSTLIGFCKISNKASNDVIAVLTDLWQEVLLPPNRKL
LAVHTRGADWKKLKKDENLRNEQKRRIYAYWHFESELKDQYHEFLKNVMQGLQTGQEHNK
NSSIVSAARLLAYAPEKEQLLLTMLVNKLGDPIAKIASKALHHLSEVAQKHPNMCGVIVA
EAEKLLFRNNISERAQHFALCFLSSIAPSGRPEVCTKLVNICFALFKVLVQKGAVNNRTM
QAILRCLQKAIVEAKPAKDSNGELLTKEMQDTIYRLVHLADIRVAVQTLGLLLQLVAVKT
EKSDRFYNALYVKLLDLNLINVGSKTAAHLLHIVHRAIHIDNHVARAQAFVKRLLQLTLY
APPHIAAGCLIVIHKLLRMRRELIGGTGASEEVEEGSKVVLPISADLDKFGSDDEEVYED
VKDEADDTKDSNPLEEKADNDVKSSASSWHHARVAATEAKVRDIDSCKYDPYHRVPAFAG
AAYALRHELLLLRQHYHPTVQVFAEQILQQSRIDYYGDPLRDFGLPHFLERFAFKNPKKL
EASQAAENATVAHKRYMAHGARGRPVKSLTKANCTEDEMFIFNFLEHKRRQAEIVAQNKK
QKEIKKDAAEEGDDGEAGEEYLKEGEVDDDEFEAYLDGYFGKKFKEGVDEEQDEEELNFL
QELGGEIKKDKSKDKKKKKQSDKAEDEMDDIDDDWGDDDLAEDEDDEIEGEDQSDDETGS
IDLQPLDDDDDDDDDDDDEGSISEGGPGDSDSSDAPESPDEEDDDDEDAPPRSKKSRKDS
TDMVGGRSFAKTLKQSHDMSSLFAAADDFSSLLEETAKVKGQGTSNAVFNKDKSSDKQLK
WEENRRSNSKSYKGKKFAGKPAAKGGRPQKAGKKRKH
```

Overall % of identity 32

PROTEIN ALIGNMENT CEBPZ and Drosophila NOC1

Query: RecName: Full=CCAAT/enhancer-binding protein zeta; AltName: Full=CCAAT-box-binding transcription factor; Short=CBF; Short=CCAAT-binding factor [Homo sapiens] Query ID: Q03701.3 Length: 1054

RecName: Full=Nucleolar complex protein 1

Sequence ID: Q9VTE6.3 Length: 1174

Range 1: 280 to 936

Score:309 bits(791), Expect:2e-92,

Method:Compositional matrix adjust.,

Identities:214/669(32%), Positives:339/669(50%), Gaps:54/669(8%)

Journal of Cell Science • Supplementary information

```
Query  225   NSQKGASSTWMKAIVSSGTLGDRMAAMILLIQDDAVHTLQFVETLVNLVK--KKGSKQQC  282
             N +  + + W++  +  GT  DR  A  LL+  + +  L+ + TL+   K   K S
Sbjct  280   NKRNPSDARWLQTALHKGTAKDRANAGALLVTSNPLGNLEALSTLIGFCKISNKASNDVI  339

Query  283   LMALDTFKELLITDLLPDNRKLRIFSQRPFD--KLEQLSSGNKDSRDRRLILWYFEHQLK  340
              +  D ++E+L    LP NRKL     R  D  KL++  +   + + R    W+FE +LK
Sbjct  340   AVLTDLWQEVL----LPPNRKLLAVHTRGADWKKLKKDENLRNEQKRRIYAYWHFESELK  395

Query  341   HLVAEFVQVLETLSHDTLVTTKTRALTVAHELLCNKPEEEKALLVQVVNKLGDPQNRIAT  400
                 EF++ +           K  ++  A  LL   PE+E+ LL  +VNKLGDP  +IA+
Sbjct  396   DQYHEFLKNVMQGLQTGQEHNKNSSIVSAARLLAYAPEKEQLLLTMLVNKLGDPIAKIAS  455

Query  401   KASHLLETLLCKHPNMKGVVSGEVERLLFRSNISSKAQYYAICFLNQMALSHEESELANK  460
             KA H L  +  KHPNM GV+  E E+LLFR+NIS +AQ++A+CFL+ +A S    E+  K
Sbjct  456   KALHHLSEVAQKHPNMCGVIVAEAEKLLFRNNISERAQHFALCFLSSIAPSG-RPEVCTK  514

Query  461   LITVYFCFFRTCVKKKDVESKMLSALLTGVNRAYPYSQTGDDK-----VREQIDTLFKVL  515
             L+ + F  F+  V+K  V ++ + A+L  + +A   ++   D       +E  DT+++++
Sbjct  515   LVNICFALFKVLVQKGAVNNRTMQAILRCLQKAIVEAKPAKDSNGELLTKEMQDTIYRLV  574

Query  516   HIVNFNTSVQALMLLFQVMNSQQTISDRYYTALYRKMLDPGLMTCSKQ--AMFLNLVYKS  573
             H+ +   +VQ L LL Q++  +   SDR+Y ALY K+LD  L+     +  A  L++V+++
Sbjct  575   HLADIRVAVQTLGLLLQLVAVKTEKSDRFYNALYVKLLDLNLINVGSKTAAHLLHIVHRA  634

Query  574   LKADIVLRRVKAFVKRLLQVTCQQMPPFICGALYLVSEILKAKPGLRSQLDDHPESDDEE  633
             +  D  + R +AFVKRLLQ+T    P    G L ++ ++L+ +  L        E ++
Sbjct  635   IHIDNHVARAQAFVKRLLQLTLYAPPHIAAGCLIVIHKLLRMRRELIGGTGASEEVEEGS  694

Query  634   NFIDANDDEDMEKFTDADKETEIVKKLETEETVPETDVETK-----KPEVASWVHFDNLK  688
               +       D++KF   D+E      K E ++T      +E K     K   +SW H
Sbjct  695   KVV-LPISADLDKFGSDDEEVYEDVKDEADDTKDSNPLEEKADNDVKSSASSWHHARVAA  753

Query  689   GGKQLN-----KYDPFSRNPLFCGAENTSLWELKKLSVHFHPSVALFAKTILQGNYIQYS  743
                ++       KYDP+ R P F GA       EL  L  H+HP+V +FA+ ILQ + I Y
Sbjct  754   TEAKVRDIDSCKYDPYHRVPAFAGAAYALRHELLLLRQHYHPTVQVFAEQILQQSRIDYY  813

Query  744   GDPLQDFTLMRFLDRFVYRNPKPHKGKENTDSVVMQPKRKHFIKDIRHLPVNSKEFLAKE  803
             GDPL+DF L  FL+RF ++NPK  +  +  ++ +  KR +     R PV S  L K
Sbjct  814   GDPLRDFGLPHFLERFAFKNPKKLEASQAAENATVAHKR-YMAHGARGRPVKS---LTK-  868

Query  804   ESQIPVDEVFF-------HRYYKKVAVKEKQ---KRDADEESIE-----------DVDDE  842
              +    DE+F          R  + VA  +KQ   K+DA EE  +           +VDD+
Sbjct  869   -ANCTEDEMFIFNFLEHKRRQAEIVAQNKKQKEIKKDAAEEGDDGEAGEEYLKEGEVDDD  927
Query  843   EFEELIDTF  851
             EFE  +D +
Sbjct  928   EFEAYLDGY  936
```

The common domains **CBP domain** and **NOC domain are indicated in bold,** as shown in the draw in Figure 1H.

Journal of Cell Science • Supplementary information

Human NOC2L and Drosophila NOC2 protein sequences

```
NOC2L human >sp|Q9Y3T9|1-749
MAAAGSRKRRLAELTVDEFLASGFDSESESESENSPQAETREAREAARSPDKPGGSPSAS
RRKGRASEHKDQLSRLKDRDPEFYKFLQENDQSLLNFSDSDSSEEEEGPFHSLPDVLEEA
SEEEDGAEEGEDGDRVPRGLKGKKNSVPVTVAMVERWKQAAKQRLTPKLFHEVVQAFRAA
VATTRGDQESAEANKFQVTDSAAFNALVTFCIRDLIGCLQKLLFGKVAKDSSRMLQPSSS
PLWGKLRVDIKAYLGSAIQLVSCLSETTVLAAVLRHISVLVPCFLTFPKQCRMLLKRMVI
VWSTGEESLRVLAFLVLSRVCRHKKDTFLGPVLKQMYITYVRNCKFTSPGALPFISFMQW
TLTELLALEPGVAYQHAFLYIRQLAIHLRNAMTTRKKETYQSVYNWQYVHCLFLWCRVLS
TAGPSEALQPLVYPLAQVIIGCIKLIPTARFYPLRMHCIRALTLLSGSSGAFIPVLPFIL
EMFQQVDFNRKPGRMSSKPINFSVILKLSNVNLQEKAYRDGLVEQLYDLTLEYLHSQAHC
IGFPELVLPVVLQLKSFLRECKVANYCRQVQQLLGKVQENSAYICSRRQRVSFGVSEQQA
VEAWEKLTREEGTPLTLYYSHWRKLRDREIQLEISGKERLEDLNFPEIKRRKMADRKDED
RKQFKDLFDLNSSEEDDTEGFSERGILRPLSTRHGVEDDEEDEEEGEEDSSNSEDGDPDA
EAGLAPGELQQLAQGPEDELEDLQLSEDD

NOC2 Dm >sp|Q9VIF0|1-766
MKLATKKIKTLGKSKPDLSKKKPAKDAIRKTKPQTTSETKVTPRNPKQKVAEPVKNGKTT
KKGFKKSHKEELEGLKDIDPEFYDFLKNNDKKLLDFNLLDTDDDDDEEGDEEDKEDTVTK
ESKDDEDDEEKYHKPSKDLEVASDESDFEVDEEDDAAAGGIQKITLNLLHQWEQQLGQAN
ISIDIVRKVIQAFNSALASISADGADGGENKHNAAAFKVVGAAAFNGVIQLCVIHLQPAI
IRLLGVRPNSSLPLHKHKKWVKVRGCLRYYLTDLIRLVEQVSSPNILGVLLKHLHQMAGM
VVPFSALGKTILKRLVVLWSTGDETVRVLAFLCILKITRKQQATMLNHVLKAMYLAYVRN
SKFVSPNTLPGINFMRRSLVEMFALDLNVSYQHVFLYIRQLAIHLRNAVILKKKDSFQAV
YNWQFINSLRLWADLLGASANKPQLQPLIYPLVTIATGVIRLIPTAQYFPLRFHCLQTLI
SLAKETNTYVPVLPLIVEVLKSNTFNRKHSAVSMKPVQFTCVLRLNKGQLAENGFRDEVI
EQVCGLLLEYLAHESTSLAFSDLVVPTVMAIKTYLKECRNANYARKLKQLLEKIQESARF
IEQQRGKSSVTFDIKDAQAVAAWEQQLRLKRTPLDVYYASWLKTHETKKRRQAAHTDEIN
ADYDVPKLKKLPVKTGVPVRNENGEVELFPSDSEDEGDDGLHLGSDDDDDEDVQEEEEVE
VEHPKAKKAKKEKPEKQKPRPATVEDDYDEAGGAVDIVKDLDLNEW
```

Overall % of identity 35.67

PROTEIN ALIGNMENT human NOC2L and Drosophila NOC2

```
Query: RecName: Full=Nucleolar complex protein 2 homolog; Short=Protein NOC2 homolog; AltName:
Full=NOC2-like protein; AltName: Full=Novel INHAT repressor [Homo sapiens] Query ID: Q9Y3T9.4
Length: 749

>nucleolar complex protein 2 [Drosophila melanogaster]
Sequence ID: NP_610095.1 Length: 766
>RecName: Full=Nucleolar complex protein 2 [Drosophila melanogaster]
Sequence ID: Q9VIF0.1 Length: 766
>nucleolar complex protein 2 [Drosophila melanogaster]
Sequence ID: AAF53971.1 Length: 766
>LD46269p [Drosophila melanogaster]
Sequence ID: AAL29053.1 Length: 766
>CG9246-RA [synthetic construct]
Sequence ID: AOQ10542.1 Length: 766
Range 1: 30 to 670

Score:427 bits(1097), Expect:5e-143,
Method:Compositional matrix adjust.,
Identities:235/653(36%), Positives:385/653(58%), Gaps:47/653(7%)

Query  33    ENSPQAETREAREAARSPDKPGGSPSASRRKGRAS---EHKDQLSRLKDRDPEFYKFLQE  89
             +  PQ  T E +   R+P +    P  + +  +      HK++L  LKD DPEFY FL+
Sbjct  30    KTKPQT-TSETKVTPRNPKQKVAEPVKNGKTTKKGFKKSHKEELEGLKDIDPEFYDFLKN  88

Query  90    NDQSLLNFSDSDSSEEEEGP-----------------------FHSLPDVLEEASEEEDG  126
             ND+ LL+F+  D+ ++++                         +H     LE AS+E D
Sbjct  89    NDKKLLDFNLLDTDDDDDEEGDEEDKEDTVTKESKDDEDDEEKYHKPSKDLEVASDESD-  147

Query  127   AEEGEDGDRVPRGLKGKKNSVPVTVAMVERWKQAAKQ-RLTPKLFHEVVQAFRAAVATTR  185
              E  E+ D    G++       +T+ ++ +W+Q   Q  ++  +  +V+QAF +A+A+
```

```
Sbjct  148  FEVDEEDDAAAGGIQK------ITLNLLHQWEQQLGQANISIDIVRKVIQAFNSALASIS  201

Query  186  GDQESAEANK-----FQVTDSAAFNALVTFCIRDLIGCLQKLLFGKVAKDSSRMLQPSSS  240
             D      NK     F+V  +AAFN ++  C+  L   + +LL   V  +SS  L
Sbjct  202  ADGADGGENKHNAAAFKVVGAAAFNGVIQLCVIHLQPAIIRLL--GVRPNSSLPLHKHKK  259

Query  241  PLWGKLRVDIKAYLGSAIQLVSCLSETTVLAAVLRHISVLVPCFLTFPKQCRMLLKRMVI  300
              W K+R  ++ YL   I+LV  +S   +L  +L+H+  +    + F    + +LKR+V+
Sbjct  260  --WVKVRGCLRYYLTDLIRLVEQVSSPNILGVLLKHLHQMAGMVVPFSALGKTILKRLVV  317
Query  301  VWSTGEESLRVLAFLVLSRVCRHKKDTFLGPVLKQMYITYVRNCKFTSPGALPFISFMQW  360
            +WSTG+E++RVLAFL + ++ R ++ T L  VLK MY+ YVRN KF SP  LP I+FM+
Sbjct  318  LWSTGDETVRVLAFLCILKITRKQQATMLNHVLKAMYLAYVRNSKFVSPNTLPGINFMRR  377

Query  361  TLTELLALEPGVAYQHAFLYIRQLAIHLRNAMTTRKKETYQSVYNWQYVHCLFLWCRVLS  420
            +L E+ AL+  V+YQH FLYIRQLAIHLRNA+  +KK+++Q+VYNWQ+++ L LW  +L
Sbjct  378  SLVEMFALDLNVSYQHVFLYIRQLAIHLRNAVILKKKDSFQAVYNWQFINSLRLWADLLG  437

Query  421  TAGPSEALQPLVYPLAQVIIGCIKLIPTARFYPLRMHCIRALTLLSGSSGAFIPVLPFIL  480
             +     LQPL+YPL  +  G I+LIPTA+++PLR HC++ L  L+  +  ++PVLP I+
Sbjct  438  ASANKPQLQPLIYPLVTIATGVIRLIPTAQYFPLRFHCLQTLISLAKETNTYVPVLPLIV  497

Query  481  EMFQQVDFNRKPGRMSSKPINFSVILKLSNVNLQEKAYRDGLVEQLYDLTLEYLHSQAHC  540
            E+ +   FNRK   +S KP+ F+ +L+L+   L E  +RD ++EQ+  L LEYL  ++
Sbjct  498  EVLKSNTFNRKHSAVSMKPVQFTCVLRLNKGQLAENGFRDEVIEQVCGLLLEYLAHESTS  557

Query  541  IGFPELVLPVVLQLKSFLRECKVANYCRQVQQLLGKVQENSAYICSRRQR--VSFGVSEQ  598
            + F +LV+P V+ +K++L+EC+ ANY R+++QLL K+QE++ +I  +R +  V+F + +
Sbjct  558  LAFSDLVVPTVMAIKTYLKECRNANYARKLKQLLEKIQESARFIEQQRGKSSVTFDIKDA  617

Query  599  QAVEAWEKLTREEGTPLTLYYSHWRKLRDREIQLEISGKERLE-DLNFPEIKR  650
            QAV AWE+  R + TPL +YY+ W K  + + + + +  + +  D + P++K+
Sbjct  618  QAVAAWEQQLRLKRTPLDVYYASWLKTHETKKRRQAAHTDEINADYDVPKLKK  670
```

The common domains indicated in the draw in Figure 1H are indicated in bold.

Human NOC3L and Drosophila NOC3 protein sequences

NOC3L human >sp|Q8WTT2|1-800
MKARRNKKQIPSFRKLIKTSKVKLENKLKNKQFKQQSTLKKYRKEQRKLRQAVKDAVSKK
PIPLENPKEKRPGKRIEREEEEEEEEALPLDMMDEDDLQLMKDLGQRVSFLTRDLSSSEPV
HAKKRKHERIIDKYEKIPRTLQTAPEKELIHLLPIKDKSGIIPQTREKPVTDSNKDEEDQ
EEERELEEEIIEDPIQELTIEEHLIERKKKLQEKKMHIAALASAILSDPENNIKKLKELR
SMLMEQDPDVAVTVRKLVIVSLMELFKDITPSYKIRPLTEAEKSTKTRKETQKLREFEEG
LVSQYKFYLENLEQMVKDWKQRKLKKSNVVSLKAYKGLAEVAVKSLCELLVALPHFNFHN
NIIVLIVPLMNDMSKLISEMCCEAVKKLFKQDKLGQASLGVIKVISGFVKGRNYEVRPEM
LKTFLCLRIKEVEVKKDTEDINKPKKFMTFKEKRKSLSRMQRKWKKAEEKLERELREAEA
SESTEKKLKLHTETLNIVFVTYFRILKKAQRSPLLPAVLEGLAKFAHLINVEFFDDLLVV
LHTLIESGDLSYQESLHCVQTAFHILSGQGDVLNIDPLKFYTHLYKTLFKLHAGATNEGV
EIVLQCLDVMLTKRRKQVSQQRALAFIKRLCTLALHVLPNSSIGILATTRILMHTFPKTD
LLLDSESQGSGVFLPELDEPEYCNAQNTALWELHALRRHYHPIVQRFAAHLIAGAPSEGS
GALKPELSRRSATELFEAYSMAEMTFNPPVESSNPKIKGKFLQGDSFLNEDLNQLIKRYS
SEVATESPLDFTKYLKTSLH

NOC3 Dm >sp|Q9VI82|1-822
MGTKKVKISSVKRAAHLKSKKTPLSKQQQQKQKQKRDQLKSKREQGQNIFSQKARKRDNL
AQRKKHNKLASLGLDPLEEDNEDGDDEMLENVADMLDGDDLALLQANKRKRKAKTTGEND
PDQGQSIGLERAYASDTKKEQDAQKIKLDLLPIKSRDGQIITRTTEVDYIPKPKQKKKNE
EEEEDDSEEDGDTEYEDSDDDVVNDVEAATAAPVQKLISTTDLLIARQQEIERQKYRIGI
ICSGLLEKPEDKMRNFHALYELMDEINPASRQANLMAVRKLAIISVTEIFKDILPEYRVG
QVDTKMQTLRKATLDRVTFENALLQQFKKFLQKLEQITAQVNRRGGLRTPQTVKLATVAV
QCMCDLLVAHPYFNYVQNIAQLLVYMLNCNYAEMRTAVHQCFRTVFSNDKRLEMTLFIVR
RINHLIKTKQNNVHVECITCLMGLKIKNVNLDAEKENELKQKKLESHRQRLLSLSKKERK
RRKKLTEVNRELEETRAEENKQAKHQKLTEIIKMVFTIYFRVLKNDPTSRVLSAILEGLA
EFAHVINLDFFSDLIDVLNRILEDQDELGYRERLHCVQTIFVILSGQGEVLNIDPIRFYQ
HFYRNMLAVQAGKNHDDFAIILRTLDEVLVKRRRNMSQQRLMAFMKRLLTGSLHLLHNGT
LATLGTIKQTFQLTSVLDNLLDTDTTIGSGRYDPELDDPEYCNAASTALYELALLARHYH
PTVRRMAVHIAHGVPATGEGALPTEIGKLTSHELFTQFDSTQMAFNPTIPLPKAGQPKLK
RGKHLYIRSDFKQEYGKLLQQGKVSQTKDKQTLQIDFFSALQ

Overall % of identity 35.02

PROTEIN ALIGNMENT human NOC3L and Drosophila NOC3

Query: RecName: Full=Nucleolar complex protein 3 homolog; Short=NOC3 protein homolog; AltName: Full=Factor for adipocyte differentiation 24; AltName: Full=NOC3-like protein; AltName: Full=Nucleolar complex-associated protein 3-like protein [Homo sapiens] Query ID: Q8WTT2.1 Length: 800

>nucleolar complex protein 3 [Drosophila melanogaster]

Sequence ID: NP_649710.1 Length: 822
>RecName: Full=Nucleolar complex protein 3; AltName: Full=NOC3-like protein; AltName: Full=Nucleolar complex-associated protein 3-like protein [Drosophila melanogaster]
Sequence ID: Q9VI82.1 Length: 822
>CG1234-PA, partial [synthetic construct]
Sequence ID: ACL92580.1 Length: 822
>nucleolar complex protein 3 [Drosophila melanogaster]
Sequence ID: AAF54043.1 Length: 822
>LD35257p [Drosophila melanogaster]
Sequence ID: AAK93278.1 Length: 822
>CG1234-PA [synthetic construct]
Sequence ID: AOQ14835.1 Length: 822
Range 1: 94 to 801
Score:397 bits(1021), Expect:2e-130,
Method:Compositional matrix adjust.,

Identities:255/722(35%), Positives:402/722(55%), Gaps:47/722(6%)

```
Query  90   DMMDEDDLQLMKDLGQRVSFLTRDLSSSEPVHAKKRKHERIIDKYEKIPRTLQTAPEKEL  149
            DM+D DDL L++    +     +    ++P   +    ER    Y    +  Q A +K
Sbjct  94   DMLDGDDLALLQ--ANKRKRKAKTTGENDPDQGQSIGLER---AYASDTKKEQDA-QKIK  147

Query  150  IHLLPIKDKSG-IIPQTRE-----KP---------------VTDSNKDEEDQEEERELEE  188
            + LLPIK + G II +T E     KP                    + E+  ++     E
Sbjct  148  LDLLPIKSRDGQIITRTTEVDYIPKPKQKKKNEEEEEDDSEEDGDTEYEDSDDDVVNDVE  207
```

Journal of Cell Science • Supplementary information

```
Query  189  EIIEDPIQEL-TIEEHLIERKKKLQEKKMHIAALASAILSDPENNIKKLKELRSMLMEQD  247
                 P+Q+L +  + LI R+++++ +K  I  + S +L  PE+ ++    L  ++ E +
Sbjct  208  AATAAPVQKLISTTDLLIARQQEIERQKYRIGIICSGLLEKPEDKMRNFHALYELMDEIN  267

Query  248  PDV----AVTVRKLVIVSLMELFKDITPSYKIRPLTEAEKSTKTRKETQKLREFEEGLVS  303
            P       + VRKL I+S+ E+FKDI P Y++  +    K    RK T     FE  L+
Sbjct  268  PASRQANLMAVRKLAIISVTEIFKDILPEYRVGQVDT--KMQTLRKATLDRVTFENALLQ  325

Query  304  QYKFYLENLEQMVKDWKQRK-LKKSNVVSLKAYKGLAEVAVKSLCELLVALPHFNFHNNI  362
            Q+K +L+ LEQ+     +R  L+    V L      A VAV+ +C+LLVA P+FN+  NI
Sbjct  326  QFKKFLQKLEQITAQVNRRGGLRTPQTVKL------ATVAVQCMCDLLVAHPYFNYVQNI  379

Query  363  IVLIVPLMNDMSKLISEMCCEAVKKLFKQDKLGQASLGVIKVISGFVKGRNYEVRPEMLK  422
              L+V ++N     +     +  + +F  DK  + +L +++ I+  +K +   V  E +
Sbjct  380  AQLLVYMLNCNYAEMRTAVHQCFRTVFSNDKRLEMTLFIVRRINHLIKTKQNNVHVECIT  439

Query  423  TFLCLRIKEVEVKKDTEDINKPKKFMTFKEKRKSLSRMQRKWKKAEEKLERELREAEASE  482
              + L+IK V +  + E+  K KK  + +++  SLS+ +RK +K   ++ REL E  A E
Sbjct  440  CLMGLKIKNVNLDAEKENELKQKKLESHRQRLLSLSKKERKRRKKLTEVNRELEETRAEE  499

Query  483  STEKKLKLHTETLNIVFVTYFRILKKAQRSPLLPAVLEGLAKFAHLINVEFFDDLLVVLH  542
            + + K +  TE + +VF  YFR+LK    S +L A+LEGLA+FAH+IN++FF DL+ VL+
Sbjct  500  NKQAKHQKLTEIIKMVFTIYFRVLKNDPTSRVLSAILEGLAEFAHVINLDFFSDLIDVLN  559

Query  543  TLIESGD-LSYQESLHCVQTAFHILSGQGDVLNIDPLKFYTHLYKTLFKLHAGATNEGVE  601
             ++E  D L Y+E LHCVQT F ILSGQG+VLNIDP++FY H Y+ +  + AG  ++
Sbjct  560  RILEDQDELGYRERLHCVQTIFVILSGQGEVLNIDPIRFYQHFYRNMLAVQAGKNHDDFA  619

Query  602  IVLQCLDVMLTKRRKQVSQQRALAFIKRLCTLALHVLPNSSIGILATTRILMHTFPKTDL  661
            I+L+ LD +L KRR+ +SQQR +AF+KRL T +LH+L N ++  L T +         D
Sbjct  620  IILRTLDEVLVKRRRNMSQQRLMAFMKRLLTGSLHLLHNGTLATLGTIKQTFQLTSVLDN  679

Query  662  LLDSESQ-GSGVFLPELDEPEYCNAQNTALWELHALRRHYHPIVQRFAAHLIAGAPSEGS  720
            LLD+++  GSG + PELD+PEYCNA +TAL+EL  L RHYHP V+R A H+  G P+ G
Sbjct  680  LLDTDTTIGSGRYDPELDDPEYCNAASTALYELALLARHYHPTVRRMAVHIAHGVPATGE  739

Query  721  GALKPELSRRSATELFEAYSMAEMTFNPPV---ESSNPKIK-GKFLQGDSFLNEDLNQLI  776
            GAL  E+ + ++ ELF  +   +M FNP +   ++  PK+K GK L   S   ++  +L+
Sbjct  740  GALPTEIGKLTSHELFTQFDSTQMAFNPTIPLPKAGQPKLKRGKHLYIRSDFKQEYGKLL  799

Query  777  KR  778
            ++
Sbjct  800  QQ  801
```

In bold are indicated the common domain as the draw in Figure 1H. The NOC domain is in red

Table S2. CRISPR Correlation Gene List

Available for download at
https://journals.biologists.com/jcs/article-lookup/doi/10.1242/jcs.264096#supplementary-data
